# Extraintestinal Symptoms in Irritable Bowel Syndrome Are Associated With Stress Reactivity and the Gut Microbiome in a Sex-dependent Manner

**DOI:** 10.1016/j.cgh.2025.07.026

**Published:** 2025-07-29

**Authors:** JONATHAN P. JACOBS, JENNIFER S. LABUS, TIEN S. DONG, ANDREA S. SHIN, UCLA SCORE GROUP IN IBS AND SEX DIFFERENCES, EMERAN A. MAYER, LIN CHANG

**Affiliations:** Vatche and Tamar Manoukian Division of Digestive Diseases, Department of Medicine, David Geffen School of Medicine at the University of California Los Angeles Los Angeles, California, *and* Goodman-Luskin Microbiome Center, David Geffen School of Medicine at the University of California Los Angeles Los Angeles, California, *and* G. Oppenheimer Center for Neurobiology of Stress and Resilience, David Geffen School of Medicine, University of California Los Angeles Los Angeles, California, *and* Division of Gastroenterology, Hepatology, and Parenteral Nutrition, VA Greater Los Angeles Healthcare System Los Angeles, California; Vatche and Tamar Manoukian Division of Digestive, Diseases, Department of Medicine, David Geffen School of Medicine at the University of California Los Angeles, Los Angeles, California, *and* Goodman-Luskin Microbiome Center, David Geffen School of Medicine at the University of California Los Angeles Los Angeles, California, *and* G. Oppenheimer Center for Neurobiology of Stress and Resilience, David Geffen School of Medicine, University of California Los Angeles Los Angeles, California; Vatche and Tamar Manoukian Division of Digestive, Diseases, Department of Medicine, David Geffen School of Medicine at the University of California Los Angeles, Los Angeles, California, *and* Goodman-Luskin Microbiome Center, David Geffen School of Medicine at the University of California Los Angeles Los Angeles, California, *and* G. Oppenheimer Center for Neurobiology of Stress and Resilience, David Geffen School of Medicine, University of California Los Angeles Los Angeles, California; Vatche and Tamar Manoukian Division of Digestive, Diseases, Department of Medicine, David Geffen School of Medicine at the University of California Los Angeles, Los Angeles, California, *and* Goodman-Luskin Microbiome Center, David Geffen School of Medicine at the University of California Los Angeles Los Angeles, California, *and* G. Oppenheimer Center for Neurobiology of Stress and Resilience, David Geffen School of Medicine, University of California Los Angeles Los Angeles, California; Vatche and Tamar Manoukian Division of Digestive Diseases, Department of Medicine, David Geffen School of Medicine at the University of California Los Angeles, Los Angeles, California, *and* Goodman-Luskin Microbiome Center, David Geffen School of Medicine at the University of California Los Angeles Los Angeles, California, *and* G. Oppenheimer Center for Neurobiology of Stress and Resilience, David Geffen School of Medicine, University of California Los Angeles Los Angeles, California; Vatche and Tamar Manoukian Division of Digestive Diseases, Department of Medicine, David Geffen School of Medicine at the University of California Los Angeles, Los Angeles, California, *and* Goodman-Luskin Microbiome Center, David Geffen School of Medicine at the University of California Los Angeles Los Angeles, California, *and* G. Oppenheimer Center for Neurobiology of Stress and Resilience, David Geffen School of Medicine, University of California Los Angeles Los Angeles, California

Extraintestinal symptoms are experienced by one-half of patients with irritable bowel syndrome (IBS).^1^ Many highly prevalent comorbid somatic conditions experienced by patients with IBS are more common in women, including fibromyalgia, chronic fatigue syndrome, chronic pelvic pain, and temporomandibular joint disorder. This has been suggested to reflect sex differences in stress, with prior work focusing on early life and adulthood adversity.^2,3^ Extending this concept, we have investigated whether measures of chronic stress reactivity (SR), which reflects tonic, trait-like stress responsiveness that is distinct from acute, adaptive stress responses, could identify a subgroup of women with increased comorbidity. We previously defined a high SR state by measures of self-reported ongoing stress (Perceived Stress Scale [PSS]) and neuroticism (International Personality Item Pool – Neuroticism [IPIP-N]) in IBS and ulcerative colitis (UC).^4,5^ Neuroticism was included as a measure of high emotional reactivity to stressors that is associated with IBS, in particular as a strong predictor of postinfection IBS risk.^6,7^ Supporting the relevance of SR defined in this manner, we found that high SR in IBS is associated with a history of early life adversity, somatic symptoms, microstructural and functional connectivity alterations in the brain, and whole blood gene expression signatures of sympathetic nervous system (SNS) activity and inflammation.^4^ Sex differences in somatic symptoms were moderated by SR, such that increased symptoms were only seen in women with high SR.^4^ Interestingly, we recently found that the high SR state in patients with UC in clinical remission was associated with alterations in intestinal microbiota and metabolites that predicted the risk of UC clinical flares.^5^ We hypothesized that the intestinal microbial signature of SR would similarly be associated with extraintestinal symptoms in IBS in a sex-dependent manner.

We investigated symptoms and the intestinal microbiome in a cohort of 221 adult Rome-positive participants with IBS (69 men, 152 premenopausal women) and 185 healthy controls (83 men, 102 premenopausal women) (Supplementary Table 1). Although the IBS group had more women than controls (69% vs 55%; *P* = .005), sex differences were based on within-group comparisons. IBS symptom severity was assessed by the IBS Severity Scoring System (IBS-SSS). Somatic symptoms were measured using the Complex Medical Symptom Inventory (CMSI), which assesses the presence (yes/no) rather than frequency of bodily symptoms over 12 months, including abdominal pain, particularly in populations with complex chronic conditions, and has been used as the primary measure of comorbidity in studies of visceral/somatic disorders (score scaled 0–1).^8^ To assess specifically for extraintestinal symptoms, we used a modified version of the Patient Health Questionnaire 12 (PHQ-12) somatic symptom severity scale, which includes the severity of 11 non-gastrointestinal somatic symptoms excluding menstrual pain (scored 0–22) over the past 4 weeks. Shotgun metagenomics was performed on stool collected from participants, who did not use antibiotics within the preceding 3 months or probiotics within the preceding month. Further methodological details are provided in the Supplementary Materials.

We found that participants with IBS had significantly increased somatic and extraintestinal symptoms compared with controls (Figure 1A). Moreover, women with IBS had increased CMSI and PHQ-12 compared with men with IBS (*P* = .005 and *P* = 3 × 10^−5^, respectively). No significant sex difference was seen in controls. Clustering the combined cohort by PSS and IPIP-N demonstrated high and low SR states equivalent to what we previously reported (Figure 1B).^5^ We then investigated whether sex differences in extraintestinal symptoms differed between the SR states. Consistent with our recent findings, increased CMSI and PHQ-12 were seen in women with IBS compared with men with IBS only in the high SR group (Figure 1C).^4^ Among controls, women had increased CMSI scores compared with men only in the high SR group, and both men and women showed a trend towards increased PHQ-12 with high SR. IBS symptom severity (IBS-SSS) showed a more subtle, but significant, difference by sex and SR, with higher IBS-SSS in women with high SR (Supplementary Figure 1A).

Using our previously described approach, we defined a SR microbiota (SRM) score from shotgun metagenomics sequence data.^5^ The SRM score reflected the probability of high SR estimated by a random forests classifier (area under the curve, 0.68) incorporating 17 bacterial species among the 48 that were significantly enriched or depleted with high SR (Figure 1D-E; Supplementary Figure 1B-C). The greatest contribution to the classifier was made by *Roseburia hominis*, which was depleted with high SR. The relationship of SRM to the original psychological measures of SR (PSS and IPIP-N) did not differ by sex (Supplementary Figure 1D). We then assessed the relationship of SRM score to somatic symptoms. High SRM was associated with increased CMSI (ie, symptom frequency) in women but not men in both IBS and controls (Figure 1F). Similarly, high SRM was associated with increased extraintestinal symptom severity by the PHQ-12 only in women with IBS. In contrast, SRM was not associated with increased IBS symptom severity (IBS-SSS) in either men or women, supporting that its relationship to somatic symptoms in women did not simply reflect greater IBS severity (Supplementary Figure 1E). SRM was significantly higher in participants with IBS than controls but did not differ between men and women (Figure 1E; Supplementary Figure 1F). Based upon prior studies demonstrating that *Roseburia* can alleviate visceral hypersensitivity and that lower microbial richness correlated with extraintestinal symptoms in women with IBS, we hypothesized that SR-associated microbial changes would be associated with somatic symptoms.^9,10^ Cross-sectional moderated mediation analysis was performed to assess to what degree increased somatic symptoms in IBS were mediated by this microbiota shift and whether sex had a moderating effect on this relationship. SRM was found to mediate 8% and 9% of the effect of IBS on CMSI and PHQ-12, respectively, with sex moderating these effects but not the direct effect of IBS on symptoms (Figure 1G).

These results support that microbial changes associated with high SR may contribute to increased extraintestinal symptoms in women with IBS relative to men with IBS but not the majority of somatic symptoms in IBS, which were independent of sex. Further work is required to determine whether the microbial signature is a cause or consequence of SR, including assessment of SNS activity and systemic inflammatory markers that may link SR to microbiota alterations. The association of CMSI with SR microbiota in control women but not men suggests that SR microbiota may also influence somatic symptoms in a broader population. Further studies are warranted to validate these findings in independent cohorts and elucidate sex-dependent biological mechanisms by which SR-associated microbiota may increase symptom generation in women with high SR.

## Supplementary Material

1

Note: To access the supplementary material accompanying this article, visit the online version of *Clinical Gastroenterology and Hepatology* at www.cghjournal.org, and at https://doi.org/10.1016/j.cgh.2025.07.026.

## Figures and Tables

**Figure 1.**
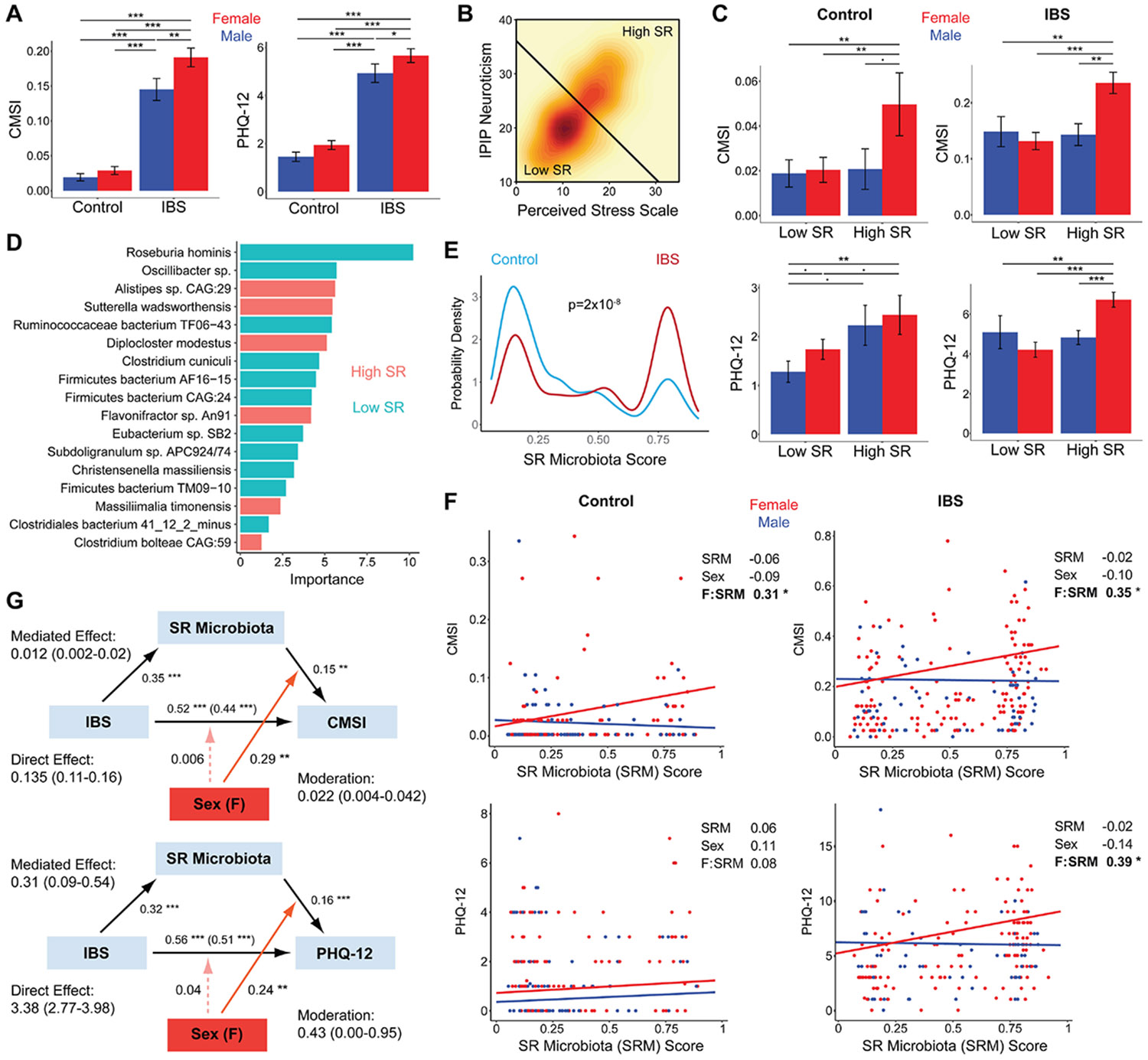
Stress reactivity-associated microbiota mediate a sex-dependent increase in extraintestinal symptoms in women with IBS. (*A*) Somatic symptoms were measured using CMSI and PHQ-12 in subjects with IBS (n = 221) and healthy controls (n = 185), stratified by sex. Significance of differences by IBS status and sex was determined by linear models adjusting for age, BMI, race/ethnicity, and diet. **P < .01; ***P < .001. (*B*) Density plot for IPIP-N and PSS in the combined cohort. A line divides the patients into high and low SR groups defined by unsupervised clustering. (*C*) CMSI and PHQ-12 in IBS and controls, stratified by SR group. Significance of differences was determined by linear models adjusting for age, sex, BMI, race/ethnicity, and diet. *P < .05; ^∙^*P* < .1. (*D*) Bacterial species contained within the random forest classifier for high vs low SR, ordered by their importance score in the classifier and colored by direction of association. (*E*) Probability density histograms of SRM score derived from the random forest classifier for subjects with IBS and controls. Significance of differences in distribution was determined by the Mann-Whitney *U* test. (*F*) Plots of CMSI and PHQ-12 by SRM score in IBS and controls, colored by sex. Effect sizes (standardized beta) are shown from general linear models including age, BMI, race/ethnicity, diet category, SRM score, sex, and the interaction of SRM with sex (F:SRM). Regression lines from the models are shown for each sex. (*G*) Cross-sectional mediation analysis was performed with IBS status as the predictor, SRM score as the mediator, and either CMSI or PHQ-12 as the outcome. Direct effects represent the effect of IBS diagnosis on CMSI or PHQ-12 in the absence of a change in SRM score. Indirect (“mediated”) effects represent the effect of IBS diagnosis on CMSI or PHQ-12 that can be explained by the increased SRM score in IBS. Direct and mediated effects are shown as difference in CMSI or PHQ-12 with 95% confidence intervals. The moderating effect of sex was also modeled to assess whether the direct and indirect effects differed between men and women. “Moderation” indicates the difference in indirect effect of IBS on CMSI or PHQ-12 (ie, attributable to increased SRM score) between women compared with men (ie, positive values indicate higher CMSI or PHQ-12 in women with IBS vs men with IBS). Diagrams show standardized betas (significance indicated by asterisks) from multivariate models with age, sex, BMI, race/ethnicity, and diet category as covariates; for the arrows connecting IBS to CMSI/PHQ-12, the standardized beta for the total effect is shown along with the direct effect in parentheses. BMI, body mass index; CMSI, Complex Medical Symptoms Inventory; IBS, inflammatory bowel syndrome; IPIP-N, International Personality Item Pool - Neuroticism; PHQ-12, Patient Health Questionnaire 12; PSS, Perceived Stress Scale; SR, stress reactivity; SRM, stress reactivity microbiota.

## Data Availability

The shotgun metagenomics sequences and associated metadata used in this study are part of several ongoing projects within our research group. Due to the active nature of these projects and our ongoing efforts in writing and publishing additional papers, we are not able to publicly release the entire dataset at this time. However, we are committed to transparency and scientific collaboration. Therefore, specific data requests can be accommodated on a case-by-case basis. We plan to submit the sequence and associated metadata to an appropriate publicly available archive once all ongoing projects are completed.
