# Supplementary material for: Extraintestinal Symptoms in Irritable Bowel Syndrome Are Associated With Stress Reactivity and the Gut Microbiome in a Sex-dependent Manner
Source: Clin Gastroenterol Hepatol. Author manuscript; Available in PMC 2026 Mar 17. (PMC12995157; doi:10.1016/j.cgh.2025.07.026)
Supplement: 1 [file NIHMS2150898-supplement-1.pdf]

Clinical Gastroenterology and Hepatology, Volume ■

Supplemental information

Extraintestinal Symptoms in Irritable Bowel Syndrome Are Associated With Stress Reactivity and the Gut Microbiome in a Sex-dependent Manner

Jonathan P. Jacobs;Jennifer S. Labus, Tien S. Dong, Andrea S. Shin;UCLA SCORE Group in IBS and Sex Differences;Emeran A. Mayer, and Lin Chang

SUPPLEMENTARY MATERIALS

Patient recruitment and assessment

All study participants were recruited from the greater Los Angeles area. Inclusion criteria for the irritable bowel syndrome (IBS) group included age 18 to 55 years and diagnosis of IBS using Rome III or IV criteria depending on year of recruitment. Exclusion criteria included menopause or perimenopause, the presence of structural gastrointestinal disease that could explain IBS symptoms, a history of malignancy within the past five years, reported gastrointestinal infection within 2 weeks before evaluation, or use of antibiotics within the preceding three months or use of probiotics within the preceding one month. Demographic data were collected by questionnaires. IBS symptom severity was assessed by the IBS Severity Scoring System (IBS-SSS, range 0-500), and presence of extraintestinal symptoms were assessed using the Complex Medical Symptom Inventory (CMSI, range 0-39 in men and 0-41 in women) and Patient Health Questionnaire 12 (PHQ-12; without three GI symptoms, range 0-22) somatic symptom severity scale.[1-3] The CMSI assesses the presence or absence of symptoms over the past 12 months, whereas the PHQ-12 evaluates the severity and bothersomeness of symptoms over the past 4 weeks. Regarding GI symptoms, the CMSI includes a single item on abdominal pain or discomfort among its 39 symptom questions for men and 41 for women (scores were scaled 0-1 to facilitate comparison between males and females). Additionally, it contains three other GI-related questions assessing heartburn, nausea, and vomiting, although these are not part of the symptom-based diagnostic criteria for IBS. In contrast, the PHQ-12 explicitly excludes all GI symptoms, making it a specific measure of extraintestinal symptom burden and somatic symptom severity. The PHQ-12 was modified to exclude the question on menstrual pain to facilitate direct comparison of scores between males and females. Stress reactivity (SR) was assessed by questionnaires measuring perceived stress (PSS, Perceived Stress Scale) and emotional reactivity to stress (International Personality Item Pool – Neuroticism [IPIP-N]).[4, 5] Diet was

categorized as either following a Standard American diet or an alternative dietary pattern (combined due to low numbers in each alternative pattern) according to a previously validated dietary pattern form.[6] Stool was collected as fresh frozen samples (n=256) or preserved in 95% ethanol (n=150).

Shotgun metagenomics sequencing

Sample aliquots were shipped to One Codex, Inc., for DNA extraction, library preparation, and sequencing. DNA extraction was performed using the DNeasy 96 PowerSoil Pro QIAcube HT extraction kit (Qiagen, Germantown, MD) according to the manufacturer's protocol. Library preparation was performed using the KAPA HyperPlus library preparation kit (Kapa Biosystems, Wilmington, MA) following the manufacturer's protocol. Sequencing was performed using an Illumina (San Diego, CA) NextSeq 2000 instrument (2x150 bp reads). Sequencing reads were processed using One Codex analysis software to assign taxonomy as previously described.[7] In brief, every sequence was compared against the One Codex database containing over 148,000 complete microbial genomes using K-mer based classification. Filtering was performed to remove suspected artifact sequences and microbial species relative abundances were estimated based upon sequencing coverage across reference genomes.

Stress Reactivity Microbiota Score

Individuals were clustered into high and low stress reactivity (SR) groups using partitioning around mediods (PAM) of manhattan distance, implemented in the pam function of the 'cluster' R package as previously described.[8] Differentially abundant microbes were identified between SR groups using multivariate general linear models implemented in MaAsLin2.[9] First, sequencing data were filtered to remove species present in <25% of samples. Relative abundances were then log-transformed prior to model fitting with age, sex, BMI, race/ethnicity, diet category, sample collection method, IBS status, and stress reactivity group as fixed effects. P-values were adjusted for multiple hypothesis testing by the

Benjamini-Hochberg method to generate q-values. Significance was set at q<0.25 for all analyses as is recommended for MaAsLin2.[9] Differentially abundant fecal microbes were inputted into the 'caret' R package to generate a random forest classifier for SR.[10] The data were split with 70% used to train the classifier and 30% used to estimate classifier performance. Contribution of each feature to classifier accuracy was assessed by variable importance scores. Features were retained in the final model if they had an importance score >2 in an initial model iteration. The accuracy of the resulting classifier was determined using the 30% test subset. Area under the receiver operating characteristic curve (AUC) with 95% confidence interval was calculated by bootstrapping using the ci.auc function in the 'pROC' R package. The random forest classifier was then applied to metagenomics data for each sample to generate a probability of belonging to the high SR group. These values ranging from 0 to 1 were used as SR Microbiota scores.

Statistics

Statistical significance was determined by linear models using the lm function in base R; standardized betas were derived from the output using the lm.beta R package. Covariates in all analyses included age, sex, BMI, race/ethnicity, and diet category. The non-parametric Mann-Whitney U test was used to compare distributions of SR Microbiota Score. Mediation analysis was performed using the 'mediation' R package.[11] First, a linear model was created using the lm function predicting the mediator (SR Microbiota (SRM) Score) from the independent variable (IBS), the moderator (sex), and their interaction (IBS:sex). Second, a linear model was created predicting the dependent variable (CMSI or PHQ-12) from the dependent variable (IBS), mediator (SRM), the moderator (sex), and their interactions (IBS:sex, SRM:sex). Age, BMI, race/ethnicity, and diet category were included as covariates in both models. Standardized betas shown in Fig. 1G were derived using the lm.beta function. The models were then inputted into the mediate function, specifying IBS as the independent variable and SRM as the mediator,

to estimate direct and indirect effects and derive confidence intervals using 1,000 simulations. The output was then passed to the test.modmed function, specifying sex as the moderator, to estimate the moderation effects and derive confidence intervals using 1,000 simulations.

References

1. Francis CY, Morris J, Whorwell PJ. The irritable bowel severity scoring system: a simple method of monitoring irritable bowel syndrome and its progress. Aliment Pharmacol Ther 1997;11:395-402.
2. Williams DA, Schilling S. Advances in the assessment of fibromyalgia. Rheum Dis Clin North Am 2009;35:339-57.
3. Spiller RC, Humes DJ, Campbell E, et al. The Patient Health Questionnaire 12 Somatic Symptom scale as a predictor of symptom severity and consulting behaviour in patients with irritable bowel syndrome and symptomatic diverticular disease. Aliment Pharmacol Ther 2010;32:811-20.
4. Cohen S, Kamarck T, Mermelstein R. A global measure of perceived stress. J Health Soc Behav 1983;24:385-96.
5. Goldberg LR, Johnson JA, Eber HW, et al. The international personality item pool and the future of public-domain personality measures. Journal of Research in Personality 2006;40:84-96.
6. Lenhart A, Dong T, Joshi S, et al. Effect of Exclusion Diets on Symptom Severity and the Gut Microbiota in Patients With Irritable Bowel Syndrome. Clin Gastroenterol Hepatol 2022;20:e465-e483.
7. Minot SS, Krumm N, Greenfield NB. One Codex: A Sensitive and Accurate Data Platform for Genomic Microbial Identification. bioRxiv 2015:027607.
8. Jacobs JP, Sauk JS, Ahdoot AI, et al. Microbial and Metabolite Signatures of Stress Reactivity in Ulcerative Colitis Patients in Clinical Remission Predict Clinical Flare Risk. Inflamm Bowel Dis 2024;30:336-346.
9. Mallick H, Rahnavard A, McIver LJ, et al. Multivariable association discovery in population-scale meta-omics studies. PLoS Comput Biol 2021;17:e1009442.
10. Breiman L. Machine Learning 2001;45:5-32.
11. Tingley D, Yamamoto T, Hirose K, et al. mediation: R Package for Causal Mediation Analysis. Journal of Statistical Software 2014;59:1 - 38.

	Controls (N=185)	IBS (N=221)	P-value
Gender			
Male	45%	31%	0.005
Female	55%	69%	
Age	28.5 (9.3)	28.4 (8.9)	0.87
BMI	25.0 (4.0)	23.9 (3.8)	0.004
Race/Ethnicity			
Non-Hispanic white	31%	47%	0.02
Hispanic	22%	20%	
African-American	6%	5%	
Asian	34%	22%	
Multiracial/other	7%	6%	
Diet			
Standard American	67%	61%	0.20
Other	33%	39%	
Bowel habit subtype			
IBS-D		33%	
IBS-C		39%	
IBS-M		21%	
IBS-U		7%	
IBS-SSS		223 (85)	
Remission (<75)		3%	
Mild (76-175)		26%	
Moderate (176-300)		52%	
Severe (>300)		19%	

Supplementary Table 1. Patient demographics and IBS severity. Continuous measures are shown as mean (standard deviation). P-values calculated by t-test or Fisher's exact test for categorical data.

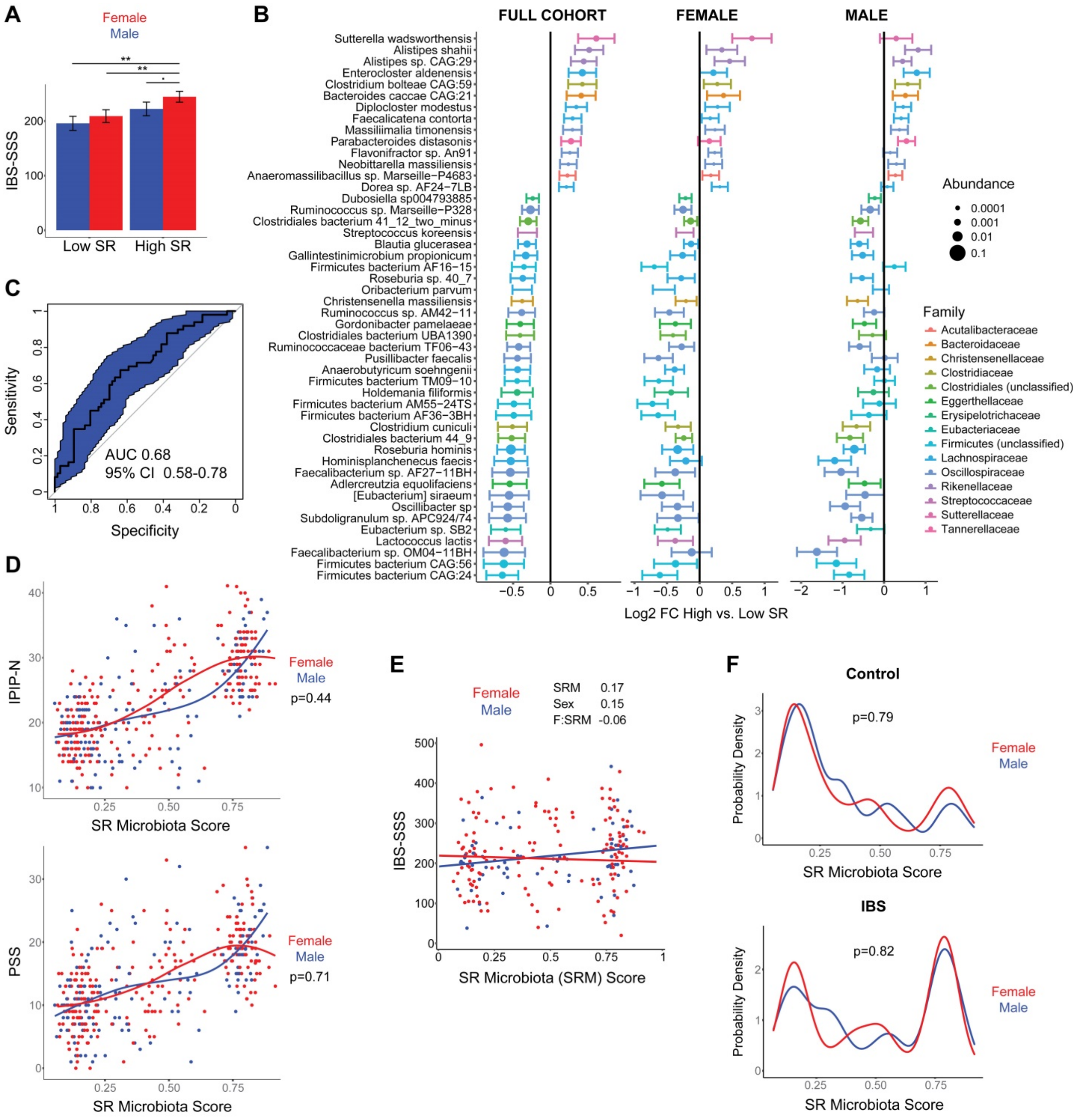

Supplementary Figure 1. A microbial signature of stress reactivity (SR) in IBS and healthy controls. (**A**) IBS symptom severity scale (IBS-SSS) is shown for IBS patients, divided by SR group and sex. Mean +/- SEM. Significance of differences was determined by linear models adjusting for age, BMI, race/ethnicity, and diet. ** p<0.01, · p<0.1 (**B**) Differentially abundant bacterial species (q<0.25 in the full cohort) between high vs. low SR are shown. Effect size is represented as the log2 fold change (FC) with 95%

confidence interval and is shown for the full cohort as well as males and females analyzed separately. Dot size corresponds to mean relative abundance of each species and color indicates bacterial family. MaAsLin2 models were adjusted for age, sex, BMI, race/ethnicity, diet category, sample collection method, and IBS status. (**C**) Receiver operating characteristics (ROC) curve for a random forest classifier differentiating high vs. low SR based upon fecal microbiome composition. Area under the curve (AUC) for the classifier is shown along with a 95% confidence interval (CI). The data were split with 70% used to train the classifier and 30% used to test the classifier and estimate AUC. (**D**) Plots of SR microbiota score (the probability of high SR estimated by the random forest classifier) by PSS and IPIP-N, colored by sex. Lines were fitted by loess. P-values were obtained from linear models including age, BMI, race/ethnicity, diet category, and IBS group as covariates. (**E**) Plot of IBS-SSS by SR Microbiota (SRM) score in IBS patients colored by sex. P-values and effect sizes (standardized beta) are shown from a linear model including age, BMI, race/ethnicity, diet category, SRM, sex, and the interaction of SRM with sex (F:SRM). Regression lines from the model are shown for each sex. (**F**) Probability density histograms of SR microbiota score derived from the random forest classifier for IBS subjects and controls, divided by sex. Significance of differences in distribution by sex was determined by the Mann-Whitney U test.
